# Multiple neuron clusters on Micro-Electrode Arrays as an in vitro model of brain network

**DOI:** 10.1038/s41598-023-42168-0

**Published:** 2023-09-20

**Authors:** Martina Brofiga, Serena Losacco, Fabio Poggio, Roberta Arianna Zerbo, Marco Milanese, Paolo Massobrio, Bruno Burlando

**Affiliations:** 1 Department of Informatics, Bioengineering, Robotics, Systems Engineering (DIBRIS), University of Genova, Genova, Italy; 2 ScreenNeuroPharm, Sanremo, Italy; 3 Department of Pharmacy (DIFAR), University of Genova, Genova, Italy; 4 Department of Pharmacy (DIFAR), Pharmacology and Toxicology Unit, University of Genova, Genova, Italy; 5 IRCCS Ospedale Policlinico San Martino, Largo Rosanna Benzi 10, 16132 Genova, Italy; 6 National Institute for Nuclear Physics (INFN), Genova, Italy

**Keywords:** Biomedical engineering, Neural circuits

## Abstract

Understanding the brain functioning is essential for governing brain processes with the aim of managing pathological network dysfunctions. Due to the morphological and biochemical complexity of the central nervous system, the development of general models with predictive power must start from in vitro brain network engineering. In the present work, we realized a micro-electrode array (MEA)-based in vitro brain network and studied its emerging dynamical properties. We obtained four-neuron-clusters (4N) assemblies by plating rat embryo cortical neurons on 60-electrode MEA with cross-shaped polymeric masks and compared the emerging dynamics with those of sister single networks (1N). Both 1N and 4N assemblies exhibited spontaneous electrical activity characterized by spiking and bursting signals up to global activation by means of network bursts. Data revealed distinct patterns of network activity with differences between 1 and 4N. Rhythmic network bursts and dominant initiator clusters suggested pacemaker activities in both assembly types, but the propagation of activation sequences was statistically influenced by the assembly topology. We proved that this rhythmic activity was ivabradine sensitive, suggesting the involvement of hyperpolarization-activated cyclic nucleotide-gated (HCN) channels, and propagated across the real clusters of 4N, or corresponding virtual clusters of 1N, with dominant initiator clusters, and nonrandom cluster activation sequences. The occurrence of nonrandom series of identical activation sequences in 4N revealed processes possibly ascribable to neuroplasticity. Hence, our multi-network dissociated cortical assemblies suggest the relevance of pacemaker neurons as essential elements for generating brain network electrophysiological patterns; indeed, such evidence should be considered in the development of computational models for envisaging network behavior both in physiological and pathological conditions.

## Introduction

The human brain is arranged as an assembly of different interconnected regions, known as *brain networks*, each hosting a dense network of synaptically-connected neurons, named *neural network*^1^. Many cell types are present within neural and brain networks (e.g., astrocytes, microglia, oligodendrocytes, pericytes, and epithelial cells), but neurons are that class of excitable cells whose electrophysiological signals can be accurately measured on different space and time scales (spikes, local field potentials, bold signals, etc.). In addition, neurons are responsible for the information transmission, as well as for the coding process thanks to the expression of complex networks^2,3^.

Neural networks in the brain should comply with two competing demands, which might also be considered as fundamental organizational principles: *functional segregation* and *integration*, enabling both the rapid extraction of information and the generation of coherent brain states^4^. As confirmed by studies reporting structural analyses of brain networks carried out on datasets describing the cerebral cortex of mammalian animal models (e.g., rat, cat, monkey), brain areas were found to be neither completely connected with each other nor randomly linked^5^; their interconnections show a specific and intricate organization. The coexistence of high degrees of local clustering and short path length is a peculiarity of modular networks^6^, nowadays considered one of the best models to support the emergence of functional segregation (associated to local assemblies) and functional integration (provided by long-range connections).

Investigating the interplay between brain functioning and the underlying connectivity is fundamental not only for basic science but also it is a prerequisite for interacting and intervening in brain networks with the ability to govern their processes, especially critical for the understanding of pathological network dysfunctions and their prevention or reversion^7,8^. This aim is still deemed extremely ambitious because current knowledge is largely insufficient. A general theory about neural and brain networks providing operationally useful results is lacking mainly because the available paradigms are burdened by the enormous morphological and biochemical complexity of the central nervous system^9^. Hence, it appears obvious that the possibility of developing general models with predictive power can only be achieved through overcoming complexity, i.e., by a reconceptualization of the system in manageable terms on the biological and mathematical sides. Theoretical studies have provided answers to important questions about brain circuitry^10^, dynamical states^11^, and their interactions^12^, nevertheless, they cannot be used to effectively interact and to appreciate possible effects of treatments for neurological pathologies.

In vitro models are a recognized compromise between the huge complexity of the entire brain and the abstracted theoretical models. In particular, the possibility to engineer brain networks paved the way to explore and find possible interplay between connectivity and expressed patterns of both spontaneous^13^ and stimulus-evoked activity^14^. The recent advancements in Micro-Electrode Array (MEA) technology have promoted the investigation of the interaction of brain networks thanks to devices with thousands of recording micro-transducers which allow mapping the propagation of the electrophysiological activity at high resolution among the different sub-populations of in vitro neuronal assemblies allowing a good tradeoff between controllability/observability and similarity to the in vivo nervous system^15^. Therefore, in vitro models have become a suitable tool to investigate brain physiological and pathological conditions^16^.

In this work, we aimed to develop a simple in vitro experimental MEA-based model to investigate a brain network consisting of four interconnected neural (cortical) assemblies. To this purpose, we designed a cross-shaped polymeric mask to separate the active area of MEA into four independent compartments. After 5 days in vitro (DIV), such a barrier was removed in order to allow a free and unconditioned neuritic growth among the clusters to realize four-neural-network systems characterized by a high degree of modularity. The emerging patterns of spontaneous electrophysiological activity were then compared with sister not-clusterized networks free to connect without physical constraint (controls). The final goal was to reveal activity patterns emerging from the interconnection of different neural networks, as compared to single networks, thus providing insight into brain information processing and posing the basis for its computational modeling.

## Materials and methods

### Polymeric devices set up on Micro-Electrode Arrays (MEAs)

**Figure 1:**
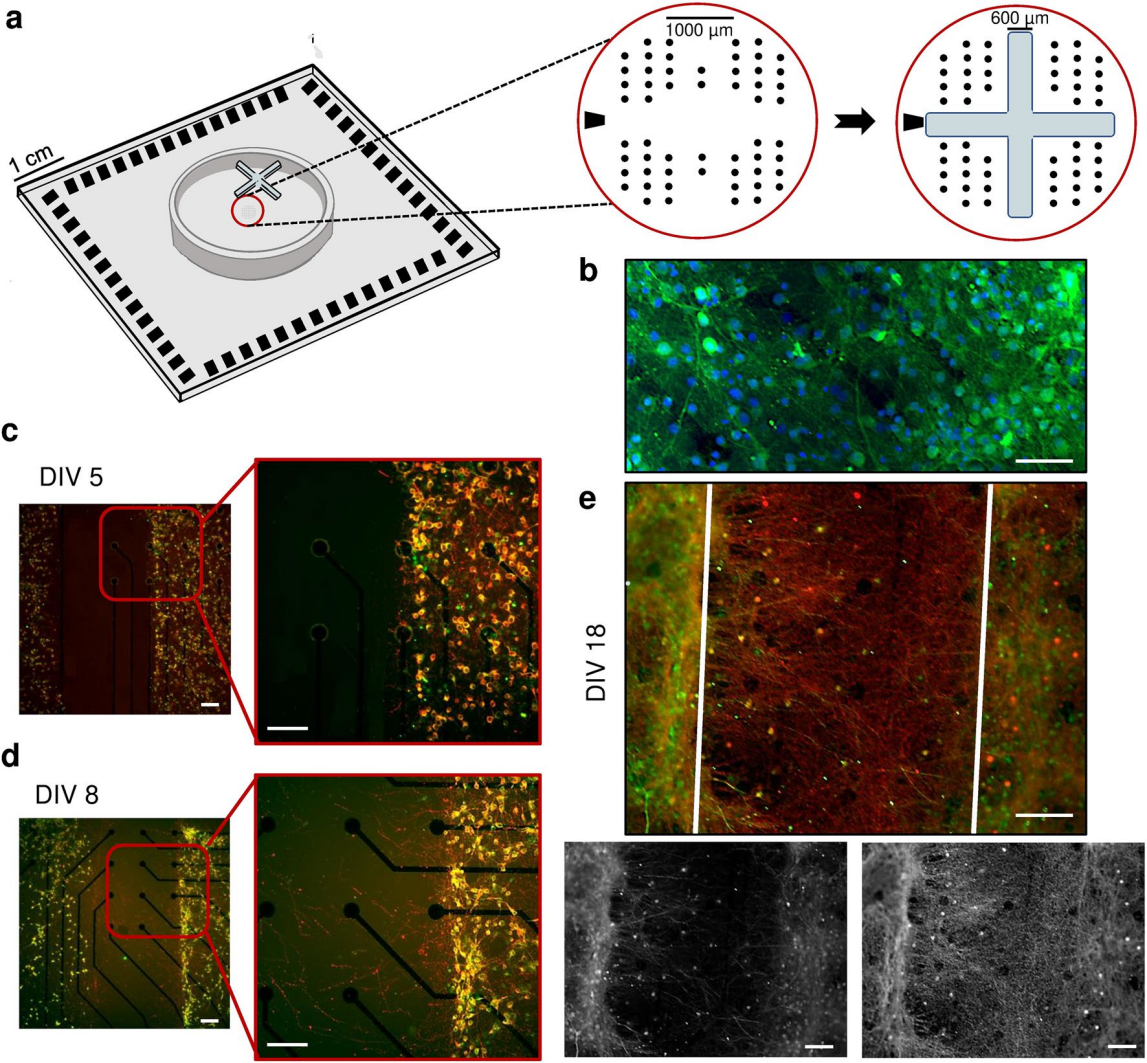
(**a**) Positioning of the cross-shaped PDMS mask on the MEA. The black dots indicate the electrode position; the black trapezoidal shape on the left individuates the reference electrode. The active area of the MEA consists of 4 electrode clusters in the corners of 1.8 mm × 1.4 mm rectangle, where each cluster consists of 13 electrodes (Ø = 30 µm), and an additional 7 electrodes are located in between clusters. (**b**) Immunofluorescence image of a representative four-neural-network (4N) at day in vitro (DIV) 18, where dendritic microtubule-associated proteins (MAP2, green) and nuclei (DAPI, blue) were labeled. Immunofluorescence images of a representative 4N culture (**c**) at DIV 5, (**d**) DIV 8, and (**e**) DIV 18. Axonal connections established between two different neural networks (clusters), where dendritic microtubule-associated protein (MAP2, green) and axon microtubule-associated protein (Tau, red) were labeled. The cross-shaped mask was removed at DIV 5. The white superimposed lines delimited the area previously occupied by the cross-shaped mask. Scale bar: 100 µm.

Polydimethylsiloxane (PDMS) constraints were used to shape the network connectivity. In particular, we realized two types of PDMS constraints: cross-shaped and circular-shaped masks. The first one consists of an equal-armed cross-shaped mask (Fig. 1a) that was used to separate the active area of MEA into four sections, realizing four-neural-network systems (4N). The cross arms were about 2 mm long, their width was about 0.6 mm, and their thickness was about 0.3 mm. In this way, until the masks were not removed, the four neuronal assemblies remained physically separate (except for the culture medium). The circular-shaped mask was used to realize one-neural-network systems (1N). It consisted of a 5 mm-diameter circle with an area of about 20 mm^2^. Both types of masks were realized by mixing PDMS prepolymer and curing agent (Sylgard 184, Sigma Aldrich) at a 10:1 (w/w) ratio, polymerized in an oven at 80 °C for 20 min. PDMS masks were sterilized in 70% ethanol for 20 min and then aligned and reversibly bounded onto planar Micro-Electrode Arrays (MEAs).

### Animals

Sprague–Dawley embryonic rats at day 18–19 (E18–19) were used. Adult animals to generate the embryos were housed and bred at the animal facility, housed at 22 ± 1 °C and 50% relative humidity, with 12 h light cycle. Pregnant female rats were anesthetized and sacrificed to obtain the E18-19 embryos. Experiments involving animals were carried out in accordance with the ARRIVE guidelines^17^ established by the European Council (EU Directive 114 2010/63/EU) and the Italian D.L. n. 26/2014 and approved by the University of Genova Ethical Committee and by the Italian Ministry of Health (Project authorization No. 2018-75f11.N.POG, 512/2015-PR and 140/2014-B-DGSAF24898). All efforts were made to minimize animal suffering and reduce the number of animals used. All methods and experiments presented in this work were performed in accordance with the relevant guidelines and regulations.

### Rat cortical neuron primary cell cultures preparation

Primary cultures of cortical rat neurons were prepared from the cerebral cortices of E18-19 embryos. Briefly, cerebral cortex was isolated from embryos under stereotaxic binocular (Nikon SMZ-2T, Japan) in cold Hank's Balanced Salt Solution (HBSS, Sigma Aldrich, W/O calcium and magnesium) medium bath at 4 °C, then meninges were removed, and the dissected tissues were transferred to freshly prepared sterile HBSS (W/O calcium and magnesium). Aliquots of cortex from 3 embryos were separated and exposed to an enzymatic digestion by trypsin solution 0.125% (Gibco Invitrogen) and DNAse 0.05% (Sigma-Aldrich), and diluted in HBSS (Sigma-Aldrich) for 18 min in water bath, at 37 °C. The enzymatic dissociation was followed by gentle mechanical trituration. Cells were re-suspended in Neurobasal medium (Gibco Invitrogen) supplemented with 2% B-27 Supplement (Gibco Invitrogen), 1% stable L-Glutamine (GlutaMAX 100x, Gibco Invitrogen) and 1% PenStrep (Penicillin–Streptomycin Solution, Gibco Invitrogen). Cells were plated on 4Q-MEAs (Multi Channel Systems, Reutlingen, Germany, MCS) pre-sterilized and pre-coated with poly-L-ornithine (100 µg/ml, Sigma Aldrich), by depositing 50 µl of cell suspension within circle masks, or 10 µl of cell suspension in each of the four sectors defined by the cross-shaped masks, obtaining a final density of 1500 cells/mm^2^. After an interval of 2.5 h, to allow for cell adhesion to substrate, aliquots of the above Neurobasal medium were added to MEA, to reach a final volume of about 1500 µl. No antimitotic drug preventing glia proliferation was added, since glia cells are known to be fundamental for the healthy development of neuronal populations^18^. Cultures were maintained at 37 °C with 5% CO_2_ and 95% humidity. Five days after plating, the cross-shaped masks were removed in order to allow the development of neural fiber extension among the 4N different neural networks (clusters). In addition, half volume of the Neurobasal medium was replaced with BrainPhys medium (StemCell Technologies) supplemented with 2% NeuroCult SM1 (StemCell Technologies), 1% GlutaMAX, and 1% PenStrep solution. Thereafter, half of the medium was changed twice a week allowing the neurons to organize into morphologically and functionally mature neural networks (Fig. 1b).

### Immunofluorescence staining

Cell cultures were fixed with 4% paraformaldehyde (Sigma-Aldrich), pH 7.4, for 15 min at room temperature. Permeabilization was achieved with phosphate buffer solution (PBS, Sigma-Aldrich) containing 0.1% Triton-X100 (Sigma-Aldrich) for 15 min at room temperature. Non-specific binding of antibodies was blocked with an incubation of 45 min in a blocking buffer solution (BBS) consisting of 3% of fetal bovine serum (FBS) in PBS. Cells were incubated with primary antibody diluted in BBS at 4 °C overnight in a humified atmosphere. The following antibodies were used: Tau (axon microtubule-associated protein, mouse monoclonal 1:500, Synaptic System), MAP2 (dendritic microtubule-associated protein, rabbit polyclonal 1:500, Synaptic System), and DAPI (nuclei, 1:1000, Synaptic System). Eventually, cultures were rinsed three times with PBS and exposed for 40 min at room temperature to the secondary antibodies: Alexa Fluor 488 (1:700, Invitrogen) and Alexa Fluor 549 (1:1000, Invitrogen), Goat anti-mouse or Goat anti-rabbit. Images were acquired with a fluorescence equipped microscope (Olympus BX-51), by using a CCD camera (Orca ER II, Hamamatsu) and Image ProPlus software (Media Cybernetic).

### Dataset and experimental protocols

The dataset used in this work consists of *n* = 16 four-network systems (4N), and n = 11 one-network systems (1N) as controls. To allow a comparison among the activations of the temporal sequences between 4N and 1N assemblies (cf., section "Clusters activation sequences"), we grouped the microelectrodes of the control networks into “virtual clusters”, each of them made up of 13 units in order to maintain the same spatial organization of the 4N ones.

In addition, to investigate the role of Hyperpolarization-activated cyclic nucleotide-gated (HCN) channels (cf. section “HCN channels are involved in the modulation of rhythmic activity”), we recorded the activity of n = 3 single-network systems (1N) to derive dose–response curves for setting the suitable concentration of the HCN inhibitor ivabradine (IVB), by the computation of the IC_50_ for mean firing rate (MFR) inhibition (cf., section "Dose–response curve (IC_50_)"). Once such a value (15 µM) was derived, we performed n = 5 recordings on 1N to evaluate the IVB effect (cf., section "HCN channels are involved in the modulation of rhythmic activity"). The total of *n* = 35 used MEAs came from 6 biological preparations, each exploited to plate both 4N and 1N assemblies. Table 1 summarizes the entire dataset used, pointing out the kind of experiment and the number of recordings. Recordings were performed at DIV 18.

**Table 1:** Summary of the dataset used in the present work.

Experiment	DIV	# MEAs	Duration of each session
Characterization of spontaneous electrophysiological activity of four-network systems (4N)	18	16	20 min
Characterization of spontaneous electrophysiological activity of one-network systems (1N)	18	11	20 min
Dose–response curve for IVB effect on MFR and IC_50_ evaluation in 1N	18	3	1 h
Evaluation of the effects induced by 15 µM IVB in 1N	18	5	10 min baseline + 10 min IVB + 10 min washout

#### Electrophysiology protocol

The spontaneous electrophysiological activity of the neural networks (both 1N and 4N) was recorded at the sampling frequency of 10 kHz using MEA2100 system (MCS). Recordings were performed outside the incubator and started about 5 min after positioning MEAs upon the heated (37 °C) amplifier location to allow cultures to recover from the thermal and mechanical stress caused by the transfer from the incubator. Each experimental session lasted 20 min. To prevent evaporation and changes in the pH of the medium, a constant slow flow of humidified gas (5% CO_2_, 20% O_2_, 75% N_2_) was maintained over the MEA. Data were acquired using MC_Rack software (MCS), while offline data analysis was performed in Matlab (The MathWorks, Natick, MS, USA).

#### Drug delivery protocol

To evaluate the effects of ivabradine (IVB, Sigma-Aldrich) on the spontaneous network activity, we increased its concentration by direct injection into the culture medium. A wide administration scale (300 nM–30 µM) with significant points in the logarithmic scale was chosen to quantify the effects on the neuronal activity. For each concentration, the electrophysiological activity was measured for 10 min. Since the increasing concentrations of IVB were sequentially applied to the culture by directly pipetting the drug solution into the medium, we discarded the first two minutes of each phase to avoid observing mechanical effects due to the administration of the compound or seeing the transient effect due to diffusion processes. Therefore, the analyses were performed on 8-min recordings for each phase (i.e., IVB concentration).

### Data analysis

#### Spike detection

In order to detect the spike occurrence, we employed the Precision Time Spike Detection (PTSD) algorithm^19^. The detection requires the definition of three parameters: I) a differential threshold for each electrode, calculated as eight times the standard deviation of the signal’s biological and thermal noise; II) the lifetime period of a spike set at 2 ms; III) the refractory period set at 1 ms. Data were not spike sorted, since, during a bursting event, a global increase of activity produces a fast sequence of spikes with different and overlapping shapes, which makes the sorting difficult and unreliable^20^.

#### Burst detection

Once the spike train was identified, the burst identification was performed by applying the string method^21^, and by setting: (I) the minimum number of spikes inside each burst (set at 5); (II) the maximum time interval that occurs between two consecutive spikes into a burst (set at 100 ms).

#### Network burst detection

The choral activity of the network, named network burst (NB), was detected by employing a self-adaptive algorithm^22^. It requires the setting of two parameters: (I) the maximum inter burst event interval for burst events within a network burst; (II) the minimum percentage of recording electrodes involved in a network burst (set at 20%).

#### Spiking and bursting statistics

In order to characterize the level of activity of the network, we computed well-known firing and bursting macroscopic metrics, starting from the Mean Firing Rate (MFR), defined as the number of spikes per second. This parameter was also used to identify the active electrodes in a MEA: if the MFR value was lower than 0.1 spikes/s, the channel was discarded from the analyses. The bursting activity was evaluated in terms of: Mean Bursting Rate (MBR) (i.e., the mean number of bursts per minute), and Burst Duration (BD) (i.e., the temporal length of bursts).

In order to define the sequences of activation of each cluster during a NB, we evaluated the instantaneous firing rate (IFR). IFR was computed by dividing the number of recorded spikes in a time-window of *Δt* = 100 ms by the bin width. Such a window was realized by means of a Gaussian kernel of width equal *Δt*. We computed the IFR of the whole network averaging the IFR of all single electrodes, and identified the time of activation of each cluster (*i*) for each NB (*k*) as the time in which the IFR reaches the maximum value during the NB:

1 $$t_{{start\left( {i,k} \right)}} = t_{{\max \left( {IFR_{i} \left( {t_{start\left( k \right)} - t_{end\left( k \right)} } \right)} \right)}}$$

where *t*_*start*(*k*)_ and *t*_*end*(*k*)_ are the starting and ending times of the *k-th* NB, respectively.

#### Dose–response curve (IC_50_)

During the delivery protocol, the MFR value of each culture was averaged on the number of active electrodes during the basal recording (initial conditions). Then, the MFR values of each experiment were normalized with respect to the corresponding values of the reference (basal) activity: such a procedure allowed to compare the MFR values of the different experiments. The variation of the normalized MFR as a function of the concentration of IVB delivered was fitted by the Hill equation (Eq. 2), a widely used model to analyze non-linear drug concentration–response relationships^23^.

2 $$MFR_{norm} ([IVB]) = MFR_{norm}^{max} + \frac{{MFR_{norm}^{min} - MFR_{norm}^{max} }}{{1 + 10^{HC(\log (IC50) - \log ([IVB]))} }}$$

In Eq. (2), $MFR_{norm}^{min}$ and $MFR_{norm}^{max}$ are the highest and the lowest normalized MFR values, and [*IVB*] is the delivered concentration of ivabradine, respectively. *HC* is the Hill coefficient, which provides the largest absolute value of the slope of the curve. Finally, the *IC*_50_ is the half maximal inhibitory concentration observed.

#### Statistical analysis

##### Descriptive and inference statistics

Descriptive and inference statistics were conducted in Matlab. Data comparisons were done by using: (I) the Mann–Whitney U test for pairwise comparison of quantitative data; (II) the Kruskal–Wallis test for multiple comparisons of quantitative data, followed by Mann–Whitney U as post-hoc test with Bonferroni’s correction for pairwise comparisons; (III) the Chi-squared test for frequency distributions among qualitative classes; (IV) the Kolmogorov Smirnov test for cumulative frequency distributions; and (V) the Wilcoxon Signed-Rank test for paired samples.

Quantitative data with extreme asymmetrical distributions were log transformed for better graphical readability in box plot diagrams. In most cases, log-transformed data did not exhibit a normal distribution according to the Anderson–Darling test. Therefore, all statistical comparisons of quantitative data were carried out with the non-parametric Mann–Whitney U and Kruskal–Wallis tests.

A significance level *α* = 0.05 was applied for all statistical tests. All the box plots reported in the present work indicate the *percentile interval* 25–75 (box), the *standard deviation* (whiskers), the mean (*square*), and the median (*line*) values.

##### Clusters activation sequences

To analyze the pattern of clusters activation during network bursts, we schematically represented the topology of cluster arrangement in the 4N assemblies as a square divided into 4 sub-squares. A similar topology was assigned to the 1N assemblies by dividing them into 4 “virtual clusters” (cf., section "Dataset and experimental protocols"). For each network burst, the sequence of the activation times of the different clusters, inferred from their IFR (cf., section "Spiking and bursting statistics"), is referred to as “activation sequence”. This latter starts from an “initiator cluster” and, for each starting cluster and sequences involving at least 3 clusters, can in theory develop along 6 different possible paths, 2 of which have no diagonal steps and are referred to as “circular paths” or “circular activation sequences”. Hence, under random propagation of the activation sequences, the probability of having a circular path is 1/3. Such a probability value was considered in the analysis of departure from randomness of activation sequence paths.

##### Monte Carlo samplings

The Monte Carlo method consists of the computational generation of a data sample under some hypothesis of randomness linked to a specific experiment. In this work, we used this method to evaluate departures from randomness in the patterns of neural network activities. We compared recorded datasets, defined as *Observed data*, with datasets generated by the Monte Carlo method, defined as *Expected data*. By using ad hoc Matlab codes, we generated random samples of cluster activation sequences and random series of consecutive activation ones for the analysis of activation sequence diversity and for the occurrence of identical consecutive activation sequences, respectively.

##### Cluster activation sequence diversity

In order to analyze the diversity of the cluster activation sequence types, we first used the Shannon diversity index^24^, defined as:

3 $$H = - \Sigma p_{i} *\ln \left( {p_{i} } \right)$$

where *p*_*i*_ is the relative frequency of the *i*th type of activation sequence. The index *H* spans between a minimum of 0 (if there is only one type of activation sequence with a relative frequency equal to 1), to a maximum of ln(*N*), where* N* is the number of different activation sequence types (if all types have the same frequency). The *H* values were then normalized by applying the Shannon equitability index^24^:

4 $$E = H/\left( {\ln N} \right)$$

which converts the Shannon diversity index to a value ranging between 0 and 1.

## Results

We explored and characterized the spontaneous activity originated by the interactions of in vitro interconnected sub-populations (4N) pointing out their role as pacemaker/dominant populations. Results are compared with cortical networks that do not display a modular connectivity (1N).

Until the neuronal sub-populations were kept isolated thanks to the presence of the PDMS cross-shaped mask (Fig. 1a), they only established a dense connectivity inside each compartment (Fig. 1b); immediately after the constraint removal (DIV 5), the space among the populations was completely empty of any kind of neuritic arborizations (Fig. 1c). Afterwards, cell bodies started to extend their arborizations in the clefts, and three days after the PDMS removal (DIV 8) bundles of neurites began to extend searching for possible targets towards the other sub-populations (Fig. 1d). At DIV 18 (the day of the recordings), the long-range connections among the clusters resulted well-structured and rich enough to guarantee an effective connectivity among the sub-populations (Fig. 1e), supporting the evidence of a physical modular connectivity.

### Spiking and bursting features are modulated by the degree of modularity

**Figure 2:**
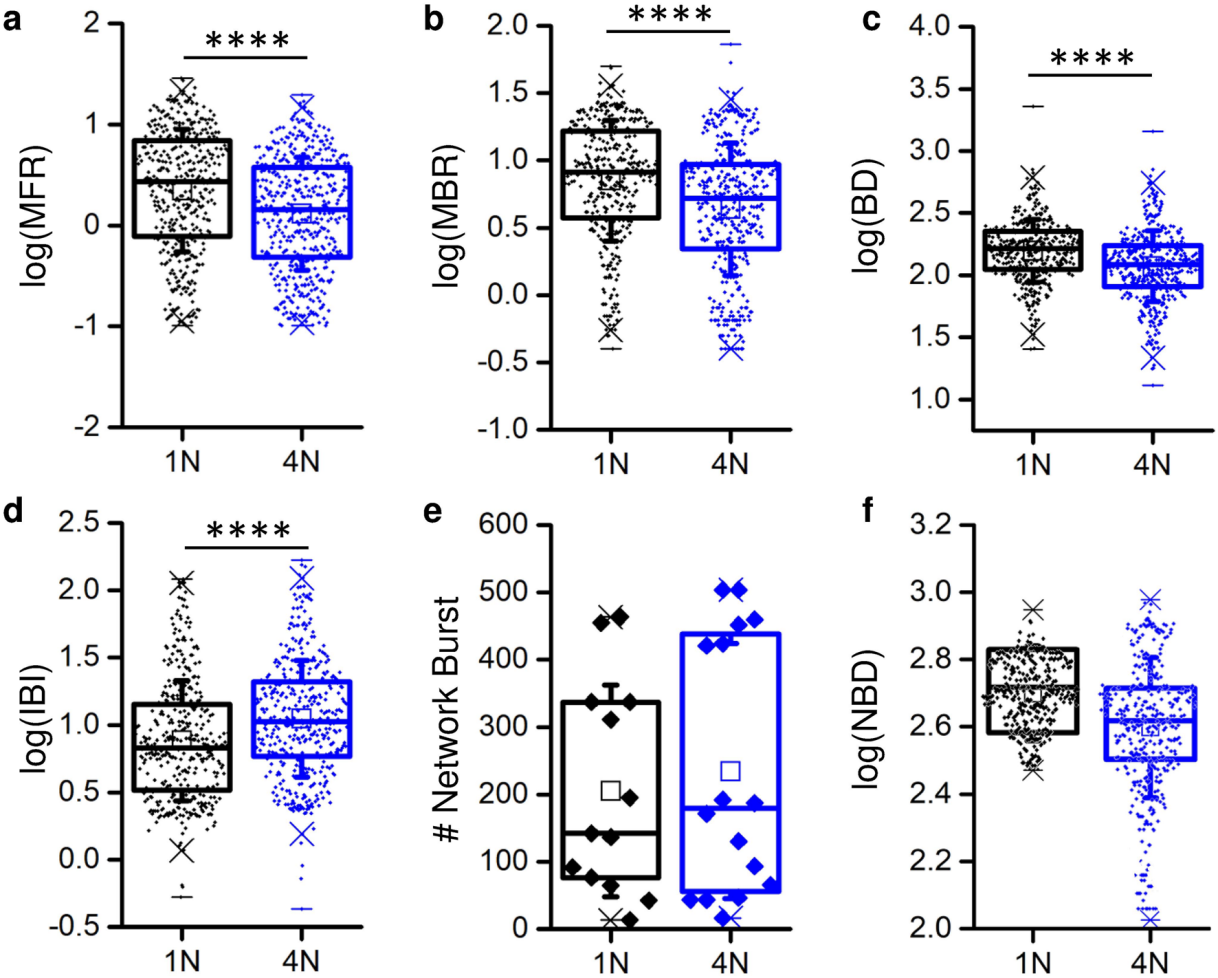
Spiking and bursting activity in one-network (1N, black) and in four-network (4N, blue) assemblies. (**a**) Mean Firing Rate (MFR), (**b**) Mean Bursting Rate (MBR), (**c**) Burst Duration (BD), (**d**) Inter Burst Interval (IBI), (**e**) Number of network bursts, (**f**) Network Burst Duration (NBD). (**a**–**d** and **f**) Each data point represents the value of a specific metric (MFR, MBR, etc.) relative to a single electrode. (**e**) Each data point is relative to a MEA. **** refers to p < 0.0001, Mann–Whitney U test.

Independently from the kind of network topology (i.e., 4N or 1N), in vitro cortical networks exhibited spontaneous electrophysiological activity, characterized by a rich repertoire of dynamics ranging from spiking and bursting signals up to the global activation of the network by means of network bursts. This behavior is consistent with both homogeneous^25^ and clustered^13^ networks coupled to MEAs. By comparing the electrophysiological activity of 1N with the one originated by 4N, significant differences were found both in spiking and bursting activity: 1N networks showed higher values of MFR than 4N (Fig. 2a), as well as MBR (Fig. 2b), and BD (Fig. 2c). Indeed, the IBI values showed an opposite trend (Fig. 2d).

Table 2 summarizes the values of the first (Q1) and third (Q3) quartiles, as well as the median, mean, and standard error of the mean, evaluated on the entire dataset of 1N and 4N assemblies. Mann–Whitney U test (since data do not follow a normal distribution, cf., Supplementary Information S1) was applied to evaluate the statistical significance between the two assemblies and the achieved *p*-values are reported in the last column of Table 2.

**Table 2:** Summary statistics of basic measurements of neural network functioning.

Parameter	Unit	Assembly	Q1	Median	Q3	Mean	S.E.M	p-values*
MFR	spikes/s	1N	0.77	2.71	6.89	4.66	0.24	4.6 × 10^−9^
		4N	0.48	1.43	3.78	2.63	0.14	
MBR	bursts/min	1N	3.75	8.21	16.5	10.7	0.42	6.5 × 10^−11^
		4N	2.23	5.23	9.36	7.44	0.37	
BD	ms	1N	112	163	225	187	7.18	1.4 × 10^−11^
		4N	81.4	122	173	147	5.61	
IBI	s	1N	3.29	6.79	14.2	13.8	0.93	8.3 × 10^−9^
		4N	5.87	10.6	21.0	18.7	1.13	
NB	NBs/min	1N	15.2	28.4	67.4	41.0	39.4	0.74
		4N	12.1	35.8	86.2	46.9	47.4	
NBD	ms	1N	392	521	646	524.5	52.6	0.09
		4N	333	415	495	431.9	44.8	

*Mann–Whitney U test.

The macroscopic metrics of the spiking and bursting statistics were modulated by the network organization of the cortical assemblies, whereas the collective activity, characterized in terms of number of network bursts (NB, Fig. 2e) and of network burst duration (NDB, Fig. 2f), did not show significant differences.

### Rhythmic patterns of activity are shaped by modularity

**Figure 3:**
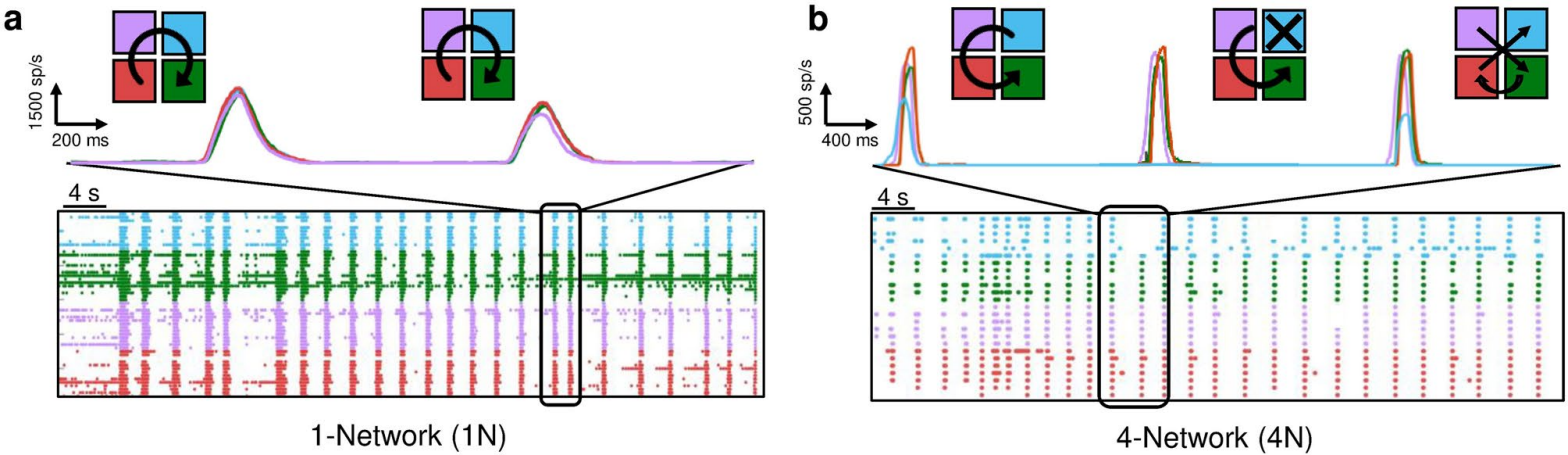
60-s periods of electrophysiological activity of representative (**a**) one-network (1N) and (**b**) four-network systems (4N). The corresponding instantaneous firing rate profiles of a 5-s time window are depicted on the top, together with a sketch of the propagation map of the cluster activation sequences. Each color identifies a “virtual” (1N) or 4N cluster, while the arrow shows the network burst propagation among the clusters. In the case of 1N, all the virtual clusters are involved, while in the case of 4N, the first and third network bursts involve all the clusters, while the second one misses the cyan cluster.

The network topology did not shape the macroscopic metrics of network bursts (Fig. 2e and 2f), conversely, their “modes” of propagation were influenced. To quantify the propagation of the electrophysiological activity, we estimated the IFR (cf.,section "Spiking and bursting statistics"). Practically, we computed the cumulative firing rate of each cluster both in 4N and 1N (considering only the active electrodes). In 1N assemblies, we grouped the electrodes into “virtual clusters” (cf., section "Dataset and experimental protocols"). Figure 3a shows a representative example of a 1N control network. The four colored traces are representative of the four “virtual clusters” used to study the activity of the network. From the visual inspection of the raster plot and the IFR profiles, a stereotyped behavior emerged, characterized by a weak modulation of the propagation of the network bursts: when this choral activity originated, it involved almost all the active electrodes. In the representative experiment depicted in Fig. 3a, the population events followed a clockwise propagation. By color-coding clusters, the network burst started in the purple cluster, then spread to the cyan, the green, and red ones. On the other hand, in the 4N representative assembly depicted in Fig. 3b, we can observe more variable and less stereotyped patterns of propagation among the clusters with respect to those observed in 1N. We also represented the IFR profiles of three network bursts: the first one shows a counterclockwise propagation involving all four interconnected populations (cyan-purple-red-green), the second network burst involves only three sub-populations (cyan is missing), and the third one involves all the clusters in a random sequence (neither clockwise nor counterclockwise).

**Figure 4:**
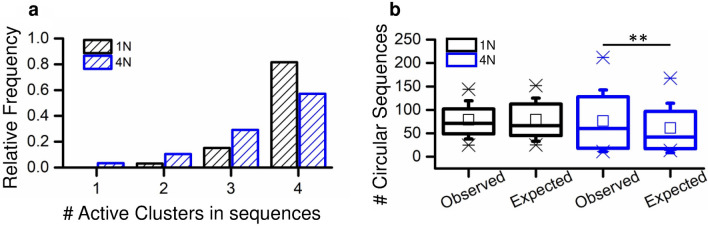
(**a**) Relative frequency of the number of recruited clusters during network bursts, evaluated by means of the IFR profile. (**b**) Box plots of the observed total numbers of circular path activation sequences in 1N (black) and 4N (blue), compared with the expected numbers obtained by considering, for each experiment, one third of the total number of sequences (under randomness, 1/3 of the recorded activation sequences should be circular path activation sequences). The comparisons of the observed and expected values by the Wilcoxon Signed-Rank test showed non-significant differences for 1N (p = 0.96) and significant ones for 4N (p = 0.005). **refers to p < 0.01. Wilcoxon Signed-Rank test.

From a quantitative point of view, we firstly considered the number of clusters involved in each activation sequence, showing that sequences of 4 clusters are significantly more likely to occur in 1N (relative frequency = 0.8, Fig. 4a, black bars), followed by sequences of 3 and 2 clusters with a relative frequency of 0.18 and 0.03, respectively. The same analysis performed on 4N networks (Fig. 4a, blue bars) showed a relative frequency of 0.58 for 4-cluster activation, and of 0.30 and 0.15 for 3 and 2 clusters, down to 0.05 in the case of a network burst confined to one cluster only. Hence, the involvement of all the clusters in the activation sequences was the most likely occurrence in both 1N and 4N. However, due to the gaps among the clusters of 4N hindering to some extent the progress of cluster activation, in the 4N a wider repertoire of propagation modes of the network burst emerged with respect to the one observed in 1N.

The peculiar spatial organization of 4N, where the sub-populations were originally separated by cross-shaped boundaries (Fig. 1a), could play a key role also in the pathway of propagation of network bursts among clusters. In particular, we could follow an entirely circular path activation sequence (CP, clockwise or counterclockwise), or a path activation sequence that cross diagonally at some point (DP). In order to understand the propagation of the observed data, we considered the activation sequences involving at least 3 clusters, and compared them with an aleatory condition (expected dataset), where the probability of having a CP is 1/3, while it is 2/3 for DP (cf., section "Clusters activation sequences"). In other words, we considered the total number of observed sequences (S) for each experiment, and defined the number of the Expected CP as 1/3 of S. By repeating this operation for all experiments, we obtained two matched samples: *Observed* circular paths and *Expected* circular paths, that were compared by the Wilcoxon Signed-Rank test. For 1N assemblies no significant variation between Observed and Expected CP was found, whereas for 4N assemblies the Observed number of CP was significantly higher than the Expected (Fig. 4b). This possibly depended on the gaps of 4N created by the cross-shaped mask (Fig. 1b), representing a higher barrier for the diagonal progress of an activation sequence. Overall, the differences observed in the lengths (Fig. 4a) and paths of activation sequences (Fig. 4b) between 1 and 4N were consistent with the different topological arrangements of these neural networks, thus validating the electrophysiological reliability of these experimental systems.

**Figure 5:**
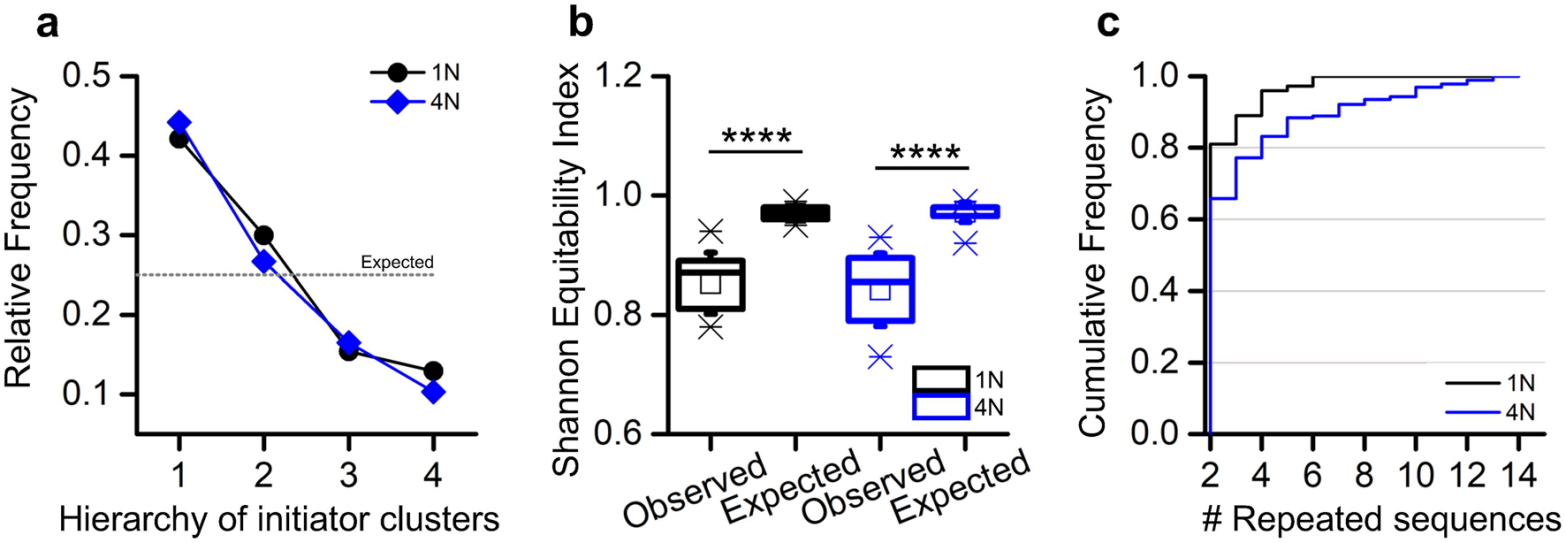
(**a**) Median relative frequencies of the starting clusters of activation sequences, obtained from the frequencies of starting clusters in each experiment. The ticks of the *x*-axis indicate the hierarchy of the initiator clusters labeling as “1″ the frequency class of the most frequent starting cluster, as ”2″ the frequency class of the second most frequent starting cluster, etc. (**b**) Box plots of the values of the Shannon equitability index, used as a measure of diversity for the activation sequences (cf., section "Statistical analysis"), derived from each experiment (Observed), and for those derived from a series of Monte Carlo samples (Expected). **** refers to p < 0.0001, Wilcoxon Signed-Rank test. (**c**) Cumulative frequency distributions derived from the total numbers of activation sequences involved in series of identical consecutive activation sequences. Significant differences were found between the 1N and 4N cumulative frequency distributions (p = 1.1** × **10^−3^, Kolmogorov–Smirnov test). Moreover, the 4N cumulative frequency distribution differs from an equivalent dataset of Monte Carlo activation sequences (p = 3.3** × **10^−4^, Kolmogorov–Smirnov test).

The different modes of propagation of the network bursting activity in 1N and 4N assemblies can be attributed to the existence of dominant clusters, which set the pace for the entire network. It has been already proved that dissociated cortical networks exhibit a subset of master or leader neurons both in silico^26^ and in vitro^27^ models, defined as a neuron that fires at the beginning of a network burst more often than expected by chance. In this work, we extended the idea of “leader” to an entire subpopulation that is able to initiate the activation sequences (initiator cluster) and evaluated their relative frequencies to assign them to frequency classes. For each 1N and 4N experiment, we identified the initiator cluster with maximum absolute frequency, assigning its relative frequency to “frequency class 1”, and repeated the operation for the next most frequent initiator cluster, until all clusters were assigned to a frequency class (classes 2 to 4). Then, we evaluated the median value of each frequency class. The results showed a significantly divergent frequency distribution both in 1N and 4N (black and blue lines in Fig. 5a, respectively, Chi-squared test p = 10^−5^) from the one that characterizes the aleatory condition (Expected) in which each cluster has the same probability of initiating a network burst (probability = 0.25, gray dotted line in Fig. 5a). From the analysis of the relative frequencies trend of the initiator clusters, two considerations arose: firstly, 1N (black line) and 4N (blue line) displayed a similar behavior; secondly, in both configurations a hierarchy was established among the clusters, with two predominant initiator clusters (i.e., clusters with a higher frequency than the expected one).

In order to investigate whether 1N and 4N models exhibit repetitive activation patterns, we compared the *Observed* activation sequences (in terms of recruitment order of clusters during a network burst) with random samples generated by Monte Carlo simulations (*Expected*), consisting of the same number of sequences (cf., section "Monte Carlo samplings"). The diversity of activation sequences in *Observed* and *Expected* samples was quantified in terms of the Shannon equitability index (*E*), which tends to 1 in random dataset, and is equal to 0 when only one sequence exists (cf., section "Cluster activation sequence diversity", Eq. 4). By deriving the Shannon equitability index values (Eq. 4) for each pair of *Observed* and *Expected* samples, we again obtained two matched series of data that were compared by the Wilcoxon Signed-Rank test. Our analysis showed that the diversity of the *Observed* activation sequences was significantly lower with respect to that of the random activation sequences (*Expected*), for both 4N (p = 3.2 × 10^−9^, Wilcoxon Signed-Rank test) and 1N (p = 3.6 × 10^−7^, Wilcoxon Signed-Rank test). In other words, some sequences were more represented than under the *Expected* (random) condition, suggesting the idea of the existence of pacemaker neurons (Fig. 5b) highly connected to the other neurons of the network forcing the emergence of repetitive activation patterns.

To further explore this behavior, we considered the occurrence of series of identical consecutive activation sequences. We defined the length of the series as the number of repeated identical consecutive sequences and computed the occurrences of the series. These two values were evaluated jointly: longer series have a higher impact than shorter ones of equal occurrence, while series that occur more frequently weight more than less frequent ones with the same length. For example, a series of 2 sequences that occurs 10 times will have a weighted absolute frequency of 2 × 10 = 20; a series of 4 sequences that occurs 5 times will have a weighted absolute frequency of 4 × 5 = 20. The corresponding cumulative frequency distribution, derived from the relative frequencies obtained from weighted absolute frequencies, highlighted a different behavior in 4N and 1N assemblies. In 1N, the longer detected series was constituted by 7 identical consecutive sequences with a frequency of 0.03. More consecutive repetitions were observed in 4N, where we detected series of up to 14 repeated sequences, these latter with a frequency of 0.01. Accordingly, the cumulative frequency distributions of 1N and 4N showed a significant difference (Fig. 5c). In addition, the 4N cumulative frequency distribution was significantly different from the one obtained from an equivalent dataset of Monte Carlo activation sequences.

### HCN channels are involved in the modulation of rhythmic activity

**Figure 6:**
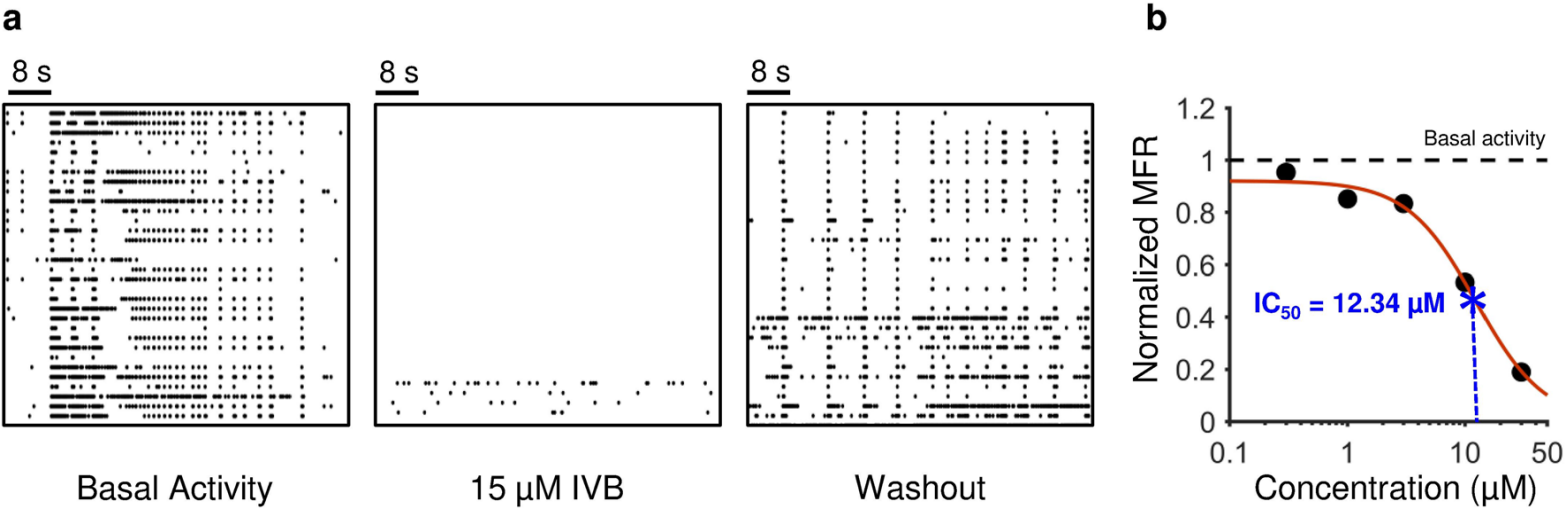
Effect of the delivery of ivabradine (IVB) on the network activity. (**a**) 60-s of electrophysiological activity of a representative cortical network before (left), during (middle), and after the delivery (right) of 15 µM IVB. (**b**) Dose–response curve of a representative cortical network obtained by delivering IVB in the range of 0.3–30 µM. The solid red line represents the Hill fitting curve (Eq. 2). The IC_50_ value is depicted with a blue star.

As a possible genesis of the network rhythmic activity, the involvement of a pool of neurons in the networks expressing hyperpolarization-activated cyclic nucleotide-gated ion channels (HCN) was hypothesized^28,29^. For this reason, we performed experiments by using ivabradine (IVB), a specific HCN channel inhibitor comparing the electrophysiological activity with and without such molecule (Fig. 6a). Since IVB acts at the single cell level, we conducted these recordings on 1N networks, since both 1N and 4N showed a rhythmic bursting activity with pacemaker features, independently on the network organization (Fig. 3). We set a concentration of IVB equal to 15 µM. This value was derived by the analysis of the dose response curve, depicted in Fig. 6b and fitted with the Hill curve of Eq. (2) (cf., section "Dose–response curve (ic50)"). We delivered increasing concentrations of IVB in the range 0.3–30 µM and recorded for each concentration the resulting electrophysiological activity (cf., section "Drug delivery protocol"). By applying the Hill fitting to the achieved MFRs, we derived an IC_50_ value of about 15 µM.

**Figure 7:**
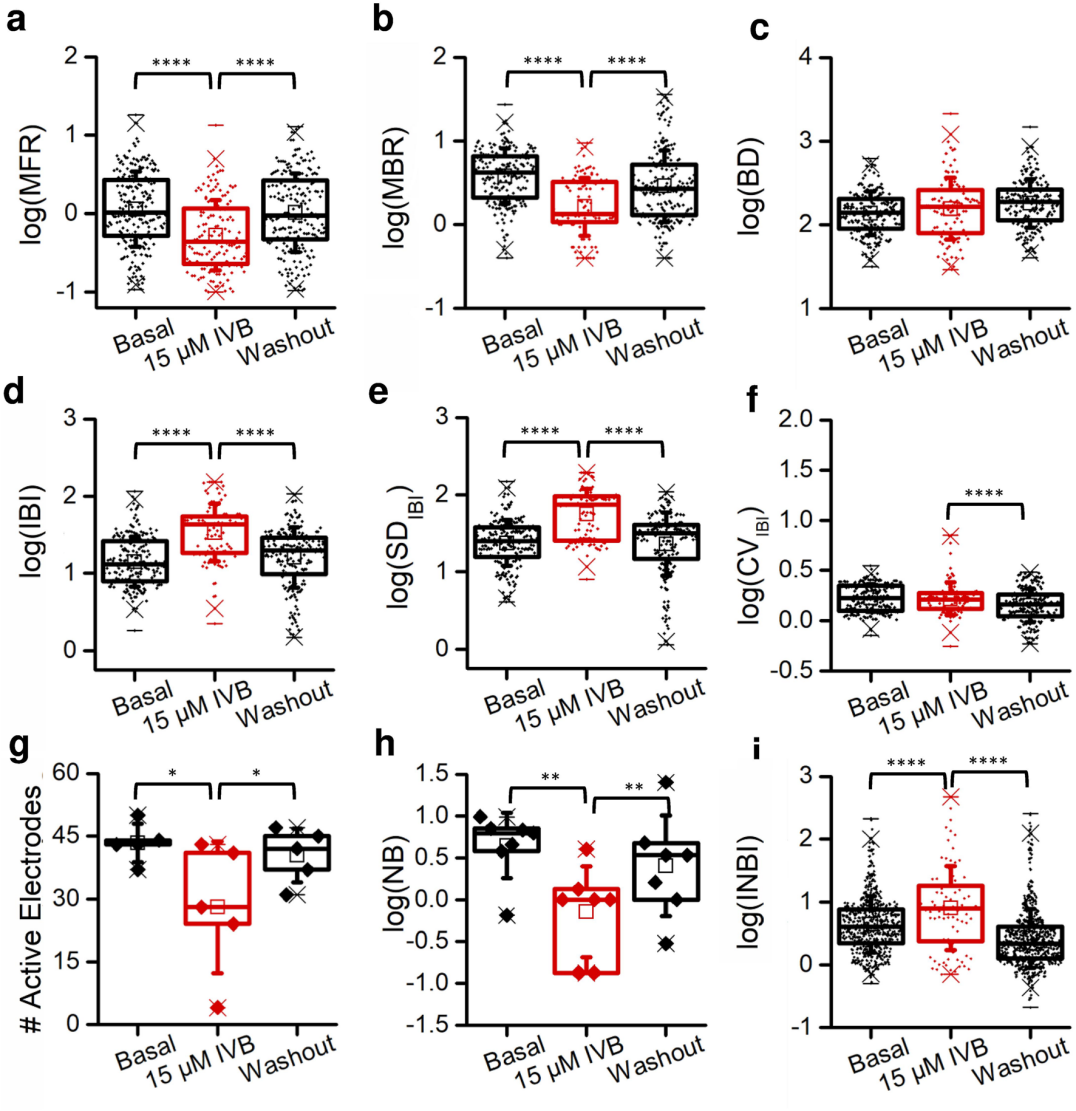
Effect of 15 µM ivabradine (IVB) on the electrophysiological activity of 1N assemblies. The boxplots show data recorded before IVB (Basal), during IVB, and after washout. (**a**) Mean Firing Rate (MFR) p = 6.8** × **10^−10^, (**b**) Mean Bursting Rate (MBR) p = 6.1** × **10^−15^, (**c**) Burst Duration (BD) p = 0.14, (**d**) Inter Burst Interval (IBI) p = 5.6** × **10^−13^, (**e**) Standard deviation of Inter Burst Interval (SD_IBI_) p = 6.6** × **10^−16^, (**f**) Coefficient of variation of the Inter Burst Interval (CV_IBI_) p = 3.5** × **10^−5^, (**g**) Number of active electrodes p = 0.03, (**h**) Number of network burst (NB) p = 6.9** × **10^−13^, (**i**) Inter network burst interval (INBI) p = 1.0** × **10^−30^. (**a**–**f** and **i**) Each data point represents the value of a specific metric (MFR, MBR, etc.) relative to a single electrode. (**g** and **h**) Each data point is relative to a MEA. *Refers to p < 0.05, ** to p < 0.01, and **** to p < 0.0001, Kruskal–Wallis test.

The effect of IVB consisted in a significant reduction in the firing and bursting frequencies (Fig. 7a,b) and in the number of active electrodes (Fig. 7g). Table 3 shows the p-values of data shown in Fig. 7 evaluated with Kruskal–Wallis since data do not follow a normal distribution (cf., Supplementary Information S2). However, the consequence of IVB delivery was transient, since, after the washout, MFR and the number of active electrodes came back to basal values, while MBR also showed the same tendency (Fig. 7a,b,g). Although in the presence of IVB the duration of the bursts remained unchanged (Fig. 7c), we observed an increase of the IBI (Fig. 7d) and of its standard deviation (Fig. 7e), but not of its variation, in the comparison between basal and IVB (Fig. 7f). Eventually, focusing on the collective behavior of the network, the delivery of IVB induced a reduction of the number of detected network bursts (Fig. 7h) and concomitantly, an increase of INBI (Fig. 7i).

**Table 3:** Probability values of statistical comparisons with data from ivabradine (IVB) experiments.

Parameter	p-values *
MFR	6.8 × 10^–10^
MBR	6.1 × 10^–15^
BD	0.13^§^
IBI	5.6 × 10^–13^
SD_IBI_	6.6 × 10^–16^
CV_IBI_	3.5 × 10^–5^
# Active electrodes	0.03
NB	6.9 × 10^–13^
INBI	1.0 × 10^–30^

*Kruskal–Wallis test.

^§^Non-significant p-value.

Hence, we can infer that IVB induced an overall reduction of network activity, and in addition it caused a rarefaction of bursts and an increase of inter-burst interval variability, i.e., overall a tendency to abolish the rhythmic activity. These data show the involvement of HCN channels in the genesis of an organized spontaneous activity of neural networks and in the network ability of realizing rhythmic patterns.

## Discussion and conclusions

In this work, we designed an in vitro experimental model to evaluate the role of the modular network organization on the patterns of electrophysiological activity exhibited by cortical networks. By exploiting physical polymeric constraints and a MEA-based set-up (Fig. 1), we engineered in vitro neuronal networks configured with a multi-network spatial organization and we explored the emerging patterns of spontaneous electrophysiological activity. This experimental model is in between a random topological configuration (the 1N configuration used as a control in this work), where neurons can grow without any constraint defining dense and un-controlled connectivity, and more patterned/organized networks where the displacement of each neuron or small assembly is finely controlled and the connectivity forced to follow well-defined rules^30,31^. Our 4N layout aims to be an in vitro configuration closer to the in vivo brain areas, where high dense connectivity exists inside the district (in our case, each sub-assembly), while among districts long-range connections are established. In vitro dissociated cortical networks display rhythmic sequences of bursts interspersed with pauses embedding isolated spikes^20^. The characterization of this dynamics has been proved following different routes (e.g., self-organized criticality, chaos theory, inferential statistics) demonstrating how cortical networks are able to generate activation patterns that are preserved in different experimental models. Rodent neuronal primary cell cultures^25^, as well as human models derived from induced pluripotent stem cells^32^ can recreate networks showing common features in the emerging electrophysiological activity. Such evidence is crucial for the aim of developing generalizable models of network activities, as the observed spontaneous activity can be considered a general reference system, independent from the recording conditions and biological source of neurons.

Our results confirm these findings, since the macroscopic statistics characterizing the spontaneous activity maintain their features also when the neuronal system is engineered as an assembly of interconnected neural networks (Fig. 2). In this kind of modular topology, the propagation of the bursting activity across the network is a distinctive element that confirms previous observations about the occurrence of burst initiation points and propagation^33^. In a previous study, initiation points have been ascribed to local inhomogeneities of the network^33^. We confirmed the occurrence of initiation points but also found that their spatial distribution is markedly non-random (Figs. 4 and 5). Hence, the choral activity of the network (network bursts) consists of a sequential activation of different sub-networks that, independent of the configuration (i.e., 1N or 4N), show non-random dominant ensembles as starting points of activation sequences, acting as “network pacemakers” (Fig. 5). In addition, some of these spontaneous sequences are more frequent than expected by chance. Remarkably, also the temporal distribution of activation sequences showed peculiar features, due to the sporadic occurrence of repetitive series of identical sequences. Interestingly, we found that only in the 4N configuration, some sequences form long, non-random, repetitive time series of identical activation sequences, i.e., realizing memory effects possibly depending on neuroplasticity. In vitro synaptic plasticity has been generally observed following high frequency tetanic stimulation of hippocampus slices^34^ or dissociated cortical networks^35^, and its effect evaluated in terms of variation of the synaptic efficacy. In our electrophysiological recordings a behavior occurred as an emergent property of spontaneous activity mainly induced by the presence of a pool of pacemaker neurons and the complex connectivity established among the sub-assemblies. Even more important, the phenomenon was significantly more pronounced in the 4N in vitro experimental model that, differently from the 1N model, has been set up creating four neuronal clusters to elementary mimic the in vivo arrangement of brain networks. Although the behavior has been unveiled exploring a simplified 4N experimental model, we believe it represents very important evidence suggesting the role of the topological connectivity that allows exhibiting complex patterns of activation. Instead, the 1N configuration that does not promote features like small-worldness^36^ or modularity^37^ (as previously observed in^38^) is less able to sustain high-order patterns of spontaneous activity. Indeed, the inferring of functional connections by exploiting correlation- or information theory-based methods could allow the quantification of these metrics and, more in perspective, the study of how such connections evolve as a function of the network development^39,40^.

The existence of network pacemakers was tested by exploring the role of hyperpolarization-activated cyclic nucleotide-gated (HCN) channels^28^. Our results showed that the inhibition of HCN currents (achieved by the exposure to ivabradine) caused a strong decline of electrophysiological activity together with the partial or total disruption of the rhythmicity and propagation of network bursting, thus highlighting a signature of pacemaker activity (Fig. 7). Intrinsic pacemaker activities related to different neurophysiological processes have been identified in many mammalian brain regions, including the respiratory central pattern generator^41^, neocortical^42^ and thalamocortical neurons^43^, medial septal neurons^44^, substantia nigra dopamine neurons^45^, and locus coeruleus noradrenergic neurons^46^. Moreover, in different of these activities a role has been ascribed to HCN channels^47,48^.

Overall, our results indicate that in vitro systems of interconnected neural networks produce a spontaneous activity that is characterized by two main features: pacemaker-dependent rhythmic activity, and spatiotemporally consolidated network patterns. These latter could also depend on neuroplasticity phenomena. They normally last on average for a few seconds, up to a maximum of about 20–25 s, i.e., falling within the temporal range reported for short-term synaptic plasticity^49^. In vitro and in vivo recordings from cortical^50^ and hippocampal^51^ assemblies revealed a reciprocal interplay between plasticity and activation patterns, since the appearance of rhythmic and persistent “modes” causes a memory stabilization. Most importantly, in our data this possibility becomes more evident when different neural networks are allowed to interplay with each other, possibly due to a better channeling of signal processing along distinct pathways, where each network would be repetitively stimulated by the upstream district and operate similarly on the downstream one, with limited randomly oriented side dispersions.

Hence, considering that there is a close similarity of our spontaneous network activity to what is generally known about in vitro neural networks, and that literature data indicate the widespread presence of pacemaker and neuroplasticity in the in vivo brain functioning^42^, we believe that pacemaker (here hypothesized and analyzed) and neuroplasticity (here only hypothesized) could represent the two basic elements characterizing the intrinsic electrophysiological activity of neuronal assemblies, both in vitro and in vivo. Regarding this view, it is remarkable that computational simulation of reconstituted cortical neural networks has been obtained by combining pacemaker activities and adaptive synapses^52^. In a set of computational works led by the group of W. Rutten, the authors proved the central role of pacemaker neurons able to drive the electrophysiological activity of the network towards a well-defined dynamical state. These neurons were supposed to be one of the most important ingredients of the network together with complex topologies and a time-dependent mechanism able to mimic the long-time constants of the network development^53^.

**Figure 8:**
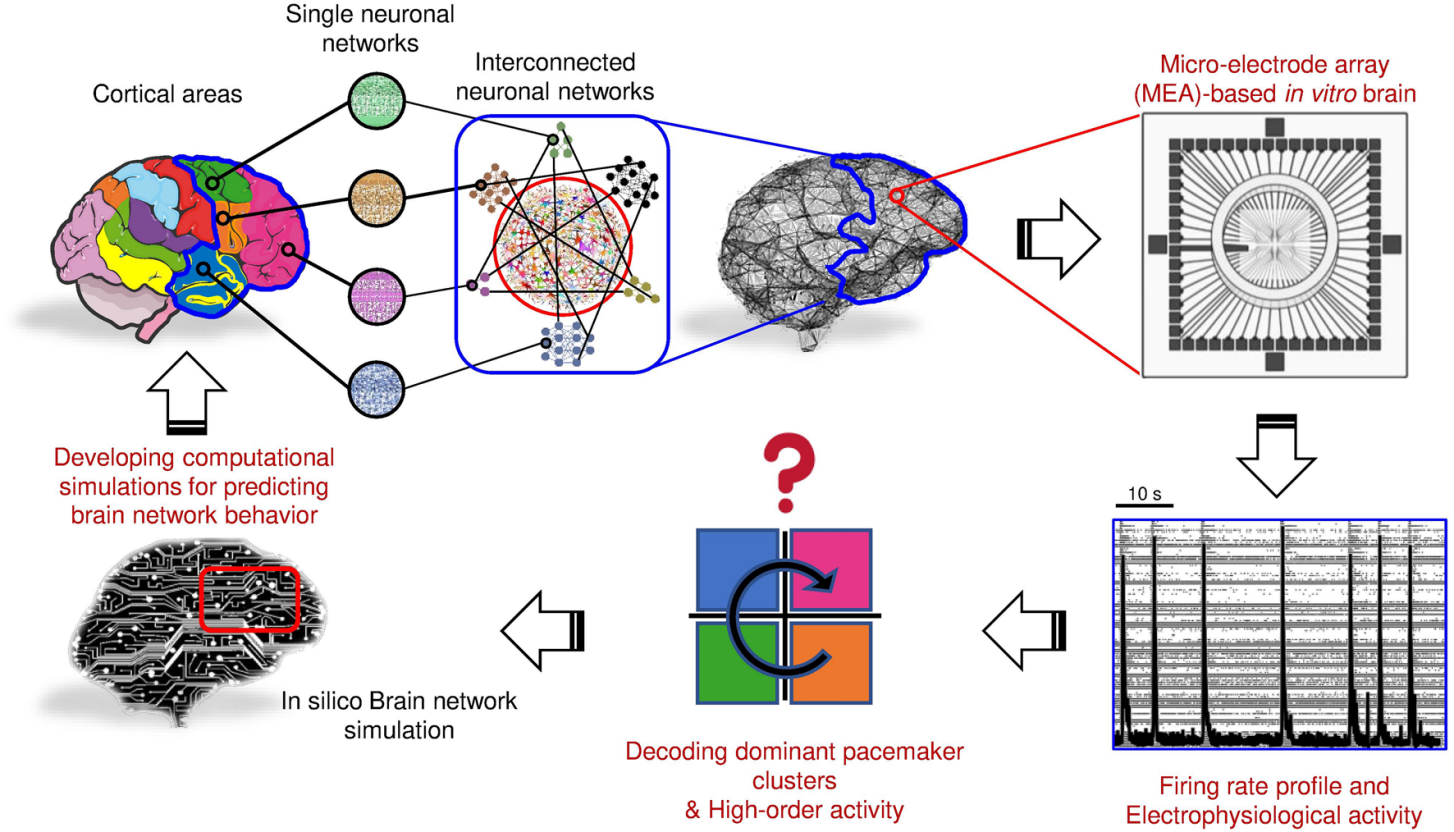
Rationale of this work and future perspectives. The main idea is to recreate interconnected neuronal networks coupled to Micro-Electrode Arrays (MEAs) to extract, examine, and quantitatively characterize their electrophysiological activity. Using this approach, we could be able to develop in silico models (*digital twins*) reproducing the observed neuronal network activity and, thus, predicting the physiological behavior of the networks. In perspective, the computational simulations could help in understanding the etiology and the progression of neurological diseases. The panels of this figure were created and drawn using and modifying images licensed under a Creative Commons Attribution and using pictures derived from the Servier Medical Art. Servier Medical Art by Servier is licensed under a Creative Commons Attribution 3.0 Unported License (https://creativecommons.org/licenses/by/3.0/).

In perspective, the achieved results provide meaning to the arrangement of brain networks, whose functioning and output could be the result of modulatory effects exerted by external inputs on the above intrinsic properties. We therefore propose engineered multi-network neuronal assemblies as in vitro simulations of brain network arrangements, and pacemaker activity and synaptic plasticity as essential elements to be considered for the decoding of their high-order activity, aimed at the development of computational simulations for predicting network behavior under physiological and pathological conditions (Fig. 8). Starting from in vitro brain network reconstruction (by exploiting the many improvements achieved in the field of the neuroengineering), continuing through the characterization of its essential distinctive features in terms of arrangement and electrophysiological functioning, and finally coming out with digital twin reproductions (in silico models), could open the way to both a deeper understanding and management of brain network disorders and the development of new generation artificial intelligence (AI) systems.

Finally, it is worth noticing that the achieved results and the 4N experimental model can also be exploited in the light of translational medicine. Many neurological diseases require structured neuronal network models to study, for example, the propagation of epileptic seizures^54^ or the damage of long-range connections among brain districts (e.g., Parkinson’s and Huntington’s diseases^55,56^). The possibility of designing a controllable and observable in vitro system will allow to study with high precision detail the impairments at the level of single neuron, single assembly, or long-range connections, as well as using it as a test bed for the evaluation of neuropharmacological drugs or specific stimulation protocols.

### Supplementary Information

Supplementary Table S1.

Supplementary Table S2.

## Data Availability

The peak trains of the entire dataset of this paper as well as the customized Matlab functions used to analyze the data have been deposited in Zenodo. The DOI of the deposited data and code reported in this paper is:10.5281/zenodo.8359135.
