# Supplementary material for: Multiple neuron clusters on Micro-Electrode Arrays as an in vitro model of brain network
Source: Sci Rep. 2023 Sep 20;13:15604. doi: 10.1038/s41598-023-42168-0 (PMC10511538; doi:10.1038/s41598-023-42168-0)
Supplement: Supplementary file 1 — Supplementary Table S1. [file 41598_2023_42168_MOESM1_ESM.pdf]

SUPPLEMENTARY INFORMATION S1

Multiple neuron clusters on Micro-Electrode Arrays as an *in vitro* model of brain network

Martina Brofiga[1,2,*], Serena Losacco[3,*], Fabio Poggio[1], Roberta Arianna Zerbo[4], Marco Milanese[4,5], Paolo Massobrio[1,6, #], and Bruno Burlando[3]

[1]Department of Informatics, Bioengineering, Robotics, Systems Engineering (DIBRIS), University of Genova, Genova, Italy

[2] ScreenNeuroPharm, Sanremo, Italy

[3]Department of Pharmacy (DIFAR), University of Genova, Genova, Italy

[4]Department of Pharmacy (DIFAR), Pharmacology and Toxicology Unit, University of Genova, Genova, Italy

[5]IRCCS Ospedale Policlinico San Martino, Largo Rosanna Benzi 10, 16132 Genova, Italy

[6]National Institute for Nuclear Physics (INFN), Genova, Italy

* These authors contributed equally to this work

# Corresponding author: paolo.massobrio@unige.it

Table S1. P-values from the Anderson-Darling Normality Test applied to raw data and log-transformed data of neural network spontaneous activity.

Parameter	1N		4N	
	**Raw data**	**Log data**	**Raw data**	**Log data**
MFR	$< 10^{-5}$	$< 10^{-5}$	$< 10^{-5}$	$< 10^{-5}$
MBR	$< 10^{-5}$	$< 10^{-5}$	$< 10^{-5}$	$< 10^{-5}$
BD	$< 10^{-5}$	$< 10^{-5}$	$< 10^{-5}$	$< 10^{-5}$
IBI	$< 10^{-5}$	$< 10^{-5}$	$< 10^{-5}$	$< 10^{-5}$
NB	0.14 §	–	$<10^{-2}$	$< 10^{-5}$
NBD	$<10^{-1}$	$<10^{-1}$	$<10^{-2}$	$<10^{-2}$

§ = not significantly different from normal distribution.
