# Supplementary material for: Multiple neuron clusters on Micro-Electrode Arrays as an in vitro model of brain network
Source: Sci Rep. 2023 Sep 20;13:15604. doi: 10.1038/s41598-023-42168-0 (PMC10511538; doi:10.1038/s41598-023-42168-0)
Supplement: Supplementary file 2 — Supplementary Table S2. [file 41598_2023_42168_MOESM2_ESM.pdf]

SUPPLEMENTARY INFORMATION S2

Multiple neuron clusters on Micro-Electrode Arrays as an *in vitro* model of brain network

Martina Brofiga[1,2,*], Serena Losacco[3,*], Fabio Poggio[1], Roberta Arianna Zerbo[4], Marco Milanese[4,5], Paolo Massobrio[1,6, #], and Bruno Burlando[3]

[1]Department of Informatics, Bioengineering, Robotics, Systems Engineering (DIBRIS), University of Genova, Genova, Italy

[2] ScreenNeuroPharm, Sanremo, Italy

[3]Department of Pharmacy (DIFAR), University of Genova, Genova, Italy

[4]Department of Pharmacy (DIFAR), Pharmacology and Toxicology Unit, University of Genova, Genova, Italy

[5]IRCCS Ospedale Policlinico San Martino, Largo Rosanna Benzi 10, 16132 Genova, Italy

[6]National Institute for Nuclear Physics (INFN), Genova, Italy

* These authors contributed equally to this work

# Corresponding author: paolo.massobrio@unige.it

Table S2. P-values from the Anderson-Darling Normality Test applied to raw data and log-transformed data of neural network activity in experiments with 15 µM ivabradine.

**Parameter**	**Basal**		**Ivabradine**		**Washout**	
	**Raw data**	**Log data**	**Raw data**	**Log data**	**Raw data**	**Log data**
MFR	$< 10^{-5}$	0.02	$< 10^{-5}$	$9 \cdot 10^{-4}$	$< 10^{-5}$	0.017
MBR	$< 10^{-5}$	$< 10^{-5}$	$< 10^{-5}$	0.002	$< 10^{-5}$	$< 10^{-5}$
BD	$< 10^{-5}$	0.69 §	$< 10^{-5}$	0.064 §	$< 10^{-5}$	0.36 §
IBI	$< 10^{-5}$	$7 \cdot 10^{-4}$	$< 10^{-5}$	0.012	$< 10^{-5}$	$< 10^{-5}$
SD_{IBI}	$< 10^{-5}$	0.01	$< 10^{-5}$	$< 10^{-5}$	$< 10^{-5}$	$< 10^{-5}$
CV_{IBI}	$< 10^{-5}$	$< 10^{-5}$	$< 10^{-5}$	$< 10^{-5}$	$< 10^{-5}$	0.11 §
# Active electrodes	0.83 §	–	0.54 §	–	0.76 §	–
NB	0.83 §	0.023	$< 10^{-5}$	0.004	$7 \cdot 10^{-4}$	0.57 §
INBI	$< 10^{-5}$	$< 10^{-5}$	$< 10^{-5}$	0.035	$< 10^{-5}$	$< 10^{-5}$

§ = not significantly different from normal distribution.
